# Metabolic diseases affect male reproduction and induce signatures in gametes that may compromise the offspring health

**DOI:** 10.1093/eep/dvaa019

**Published:** 2020-12-08

**Authors:** Sara C Pereira, Luís Crisóstomo, Mário Sousa, Pedro F Oliveira, Marco G Alves

**Affiliations:** Unit for Multidisciplinary Research in Biomedicine (UMIB), Laboratory of Cell Biology, Department of Microscopy, Institute of Biomedical Sciences Abel Salazar (ICBAS), University of Porto, Porto, Portugal; Unit for Multidisciplinary Research in Biomedicine (UMIB), Laboratory of Cell Biology, Department of Microscopy, Institute of Biomedical Sciences Abel Salazar (ICBAS), University of Porto, Porto, Portugal; Unit for Multidisciplinary Research in Biomedicine (UMIB), Laboratory of Cell Biology, Department of Microscopy, Institute of Biomedical Sciences Abel Salazar (ICBAS), University of Porto, Porto, Portugal; QOPNA & LAQV, Department of Chemistry, University of Aveiro, Aveiro, Portugal; Unit for Multidisciplinary Research in Biomedicine (UMIB), Laboratory of Cell Biology, Department of Microscopy, Institute of Biomedical Sciences Abel Salazar (ICBAS), University of Porto, Porto, Portugal

**Keywords:** epigenetic inheritance, metabolic profile, spermatozoa, obesity, diabetes

## Abstract

The most prevalent diseases worldwide are non-communicable such as obesity and type 2 diabetes. Noteworthy, the prevalence of obesity and type 2 diabetes is expected to steadily increase in the next decades, mostly fueled by bad feeding habits, stress, and sedentarism. The reproductive function of individuals is severely affected by abnormal metabolic environments, both at mechanical and biochemical levels. Along with mechanical dysfunctions, and decreased sperm quality (promoted both directly and indirectly by metabolic abnormalities), several studies have already reported the potentially harmful effects of metabolic disorders in the genetic and epigenetic cargo of spermatozoa, and the epigenetic inheritance of molecular signatures induced by metabolic profile (paternal diet, obesity, and diabetes). The inheritance of epigenetic factors towards the development of metabolic abnormalities means that more people in reproductive age can potentially suffer from these disorders and for longer periods. In its turn, these individuals can also transmit this (epi)genetic information to future generations, creating a vicious cycle. In this review, we collect the reported harmful effects related to acquired metabolic disorders and diet in sperm parameters and male reproductive potential. Besides, we will discuss the novel findings regarding paternal epigenetic inheritance, particularly the ones induced by paternal diet rich in fats, obesity, and type 2 diabetes. We analyze the data attained with *in vitro* and animal models as well as in long-term transgenerational population studies. Although the findings on this topic are very recent, epigenetic inheritance of metabolic disease has a huge societal impact, which may be crucial to tackle the ‘fat epidemic’ efficiently.

## Introduction

**Figure 1:**
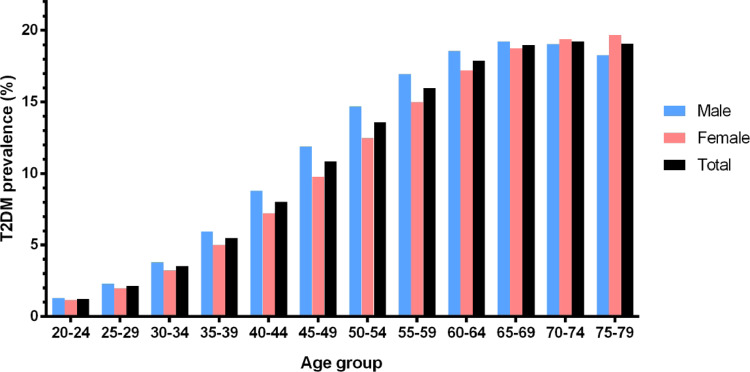
present figure for global diabetes prevalence in adults, per gender and age group. Results are expressed in percentage of the total age subjects in the age group/gender group

The prevalence of metabolic diseases has soared globally in recent decades (Fig. 1). As non-communicable diseases, they are largely caused by lifestyle, mainly diet and physical (in)activity. The fast pace characteristic of our more developed societies pushes populations toward unhealthy dietary choices highlighted by current epidemiologic data presented by the World Health Organization (WHO) rendering obesity as a global pandemic (1). However, obesity is a multifactorial disease associated with several co-morbidities, including type 2 diabetes (T2D). Taken together with their precursors, particularly overweight and prediabetes, T2D accounts for one of the main reasons for years of poor health and premature retirement worldwide. Besides, along with the negative impact on productivity, the patients suffering from those diseases also pose a significant expense for health systems highlighting metabolic diseases as a preeminent economic issue that should be taken into consideration. Several other silent co-morbidities are usually overlooked, even with new research showing that people living with metabolic diseases for part of their lives may pass metabolic signatures to the next generation. Indeed, as the prevalence of obesity and T2D is expected to increase in the upcoming decades, and more people will live with the disease for most of their active life, it is becoming very relevant to discuss how their reproduction may also contribute to this pandemic. The relationship between mother’s obesity and the metabolic phenotype of the offspring has been somewhat characterized (2) but the same is not as true for males.

Interestingly, as we witness a rise in the prevalence of metabolic diseases, fertility rates and sperm parameters are reported to decline concomitantly (3, 4). Although the specific biochemical mechanism(s) that associate metabolic disease with subfertility and infertility remains unknown, it must be highlighted that the reproductive function relies on a tightly regulated hormonal and nutritional balance (5–8). Therefore, it is not surprising that reproductive function may suffer changes accordingly with the metabolic status of the individual. More recently, it has been reported that predisposition to metabolic disease can be inherited via the paternal lineage. Interestingly, some of those reports seem like a revival of Lamarck’s theories, almost two centuries later, showing that offspring acquires adaptive and pathological features in response to environmental conditions and pressure. The truth is, those findings clearly show an interaction between environment and phenotype.

Herein, we discuss recent literature on the emerging role of metabolic status and the information conveyed in the male gamete. We have included some older works which were deemed unavoidable for the comprehension of the subject or works which presented a hallmark to the present knowledge. Our bibliographical review spanned for the first trimester of 2020, using PubMed, Scopus, and Google Scholar specialized search engines. Key search terms included: ‘spermatogenesis’, ‘obesity’, ‘mechanical dysfunctions’, ‘spermatic quality’, ‘western-diet’, ‘weight loss’, ‘bariatric surgery’, ‘obesity-related genes’, ‘epigenetic’, ‘sncRNA (small non-coding RNA)’, ‘sperm RNA’, ‘sperm epigenome’, ‘transgenerational study’, ‘epigenetic inheritance’, ‘metabolic signatures’, ‘diabetes miRNA (micro RNA)’, ‘male gamete’. MeSH (Medical Subject Headings) terms were applied to our search whenever possible.

## Overweight and Obesity: Etiology and Acquired Profile

Obesity results from a disturbance between energy intake and expenditure, inducing excessive fat accumulation in the adipose tissue. In humans, there are two major types of adipose tissue: white and brown adipose tissue. Brown adipose tissue uses energy to maintain body temperature, in a process called thermogenesis. This tissue is very abundant in newborn babies and though there is a decrease in its deposition with aging, it is still possible to find in adults (9). The white adipocytes are the most abundant adipocytes in humans and its main function is to store energy. There are many types of white adipose tissue, classified by its location, but the white intra-abdominal adipose tissue seems to be the most relevant to the development of metabolic abnormalities (10). Furthermore, the white adipocyte tissue also provides mechanical protection and thermal isolation to the body.

Fat accumulation is determined by the balance between fat synthesis, a process known as lipogenesis; and fat breakdown, a process known as lipolysis, which also includes fatty acid oxidation. Lipogenesis is a process responsive to diet and stimulated when high levels of free fatty acid and glucose can be found in the bloodstream (11). Similarly, a state of lack of energy induces lipolysis and fatty acids oxidation (12). There is no linear correlation between the body mass index (BMI), fat mass and adipocyte size. Furthermore, different adipose depots respond to weight-gain differently, mainly through white adipose tissue hyperplasia (increase in cell number), and/or hypertrophy (enlargement of individuals cells) (13, 14). When enlarged adipocytes are not able to expand anymore due to triglyceride saturation, rupture of the cells occurs and there is macrophage invasion, increased release of pro-inflammatory adipokines, and decrease in anti-inflammatory adipokines. Thus, obesity is usually associated with a chronic inflammation state (15). Furthermore, the saturation of adipocytes leads to the deposition of triglycerides in undesirable places, such as vital organs, which can lead to serious complications.

The excessive accumulation of fat and obesity is socially discussed as the result of lack of exercise, a sedentary lifestyle, and diets rich in fats. However, this phenomenon is not exclusively behavioral: recent studies show that the development of obesity has an environmental contribution. Indeed, some environmental compounds are even classified as obesogens, which describe a class of chemical compounds that can enhance adipogenesis and promote lipid accumulation. The exposure to these compounds, usually through diet, impairs energy metabolic pathways, and disrupts signaling pathways (16). Obesogens seem to have great stability and a large portion of these compounds are lipophilic. Furthermore, the chemical structure of these compounds is similar to that of several physiological regulators, including hormones. This means that obesogens have the potential to be powerful endocrine disruptors. These effects are reflected in the reproductive systems of both, males and females (17, 18). Studies in families involving twins and adopted children have provided compelling evidence that adiposity is highly heritable and that the development of obesity has a strong genetic contribution (19, 20). Those reports showed that children born from obese parents have a genetic predisposition to develop metabolic disorders. Later, obesity was found to be strongly related to genetics and epigenetics causes (21) illustrating that the metabolic profile associated with obesity and overweight can be inherited.

## Obesity and the Mechanical Dysfunctions That Hamper Sexual Activity

Overweight and obese individuals (both male and female) have a high frequency of problems related to the performance during sexual intercourse, including lack of sexual desire and poorer sexual performance (22). Also, obese men are commonly associated with an increased risk of developing erectile dysfunction and penile vascular impairment. By definition, these mechanical and psychic dysfunctions are associated with an abnormal function of the penile vasculature, due to aging or comorbid diseases (23). Naturally, risk factors for the development of cardiovascular diseases are also associated with erectile dysfunction. These risk factors include lipids abnormalities, hypertension, diabetes mellitus (DM), and obesity (24). As expected, metabolic disorders have been inversely correlated with penile arterial flow rates (25). This finding suggests that penile arterial smooth muscle relaxation capacity is altered by the metabolic profile. More specifically, it impairs endothelium-dependent vasorelaxant substances production and decreases distensibility (24), which is reflected in erectile dysfunction. When erectile dysfunction becomes too severe, the development of other mechanical disorders, such as premature ejaculation, can occur. However, it has not yet been settled the degree of interrelation between erectile dysfunction and premature ejaculation (26). To further stimulate the discussion, premature ejaculation was reported to have an inverse relationship with obesity (27). These findings highlight the complex relationship between sexual and metabolic disorders.

Lifestyle changes and weight loss are associated with an improvement in sexual function in about one-third of the obese men with erectile dysfunction, as reported by Esposito *et al.* (28). This study was conducted in 55 obese men which achieved a loss of 10% of their initial weight by exercise and healthy food choices. Also, obesity is often associated with impaired emotional health and negative body image. Along with the social stigmatization associated with erectile dysfunction, obese/overweight individuals feel discouraged to seek medical help (22).

## Multifactorial Effect of Obesity in Sperm Quality

**Figure 2:**
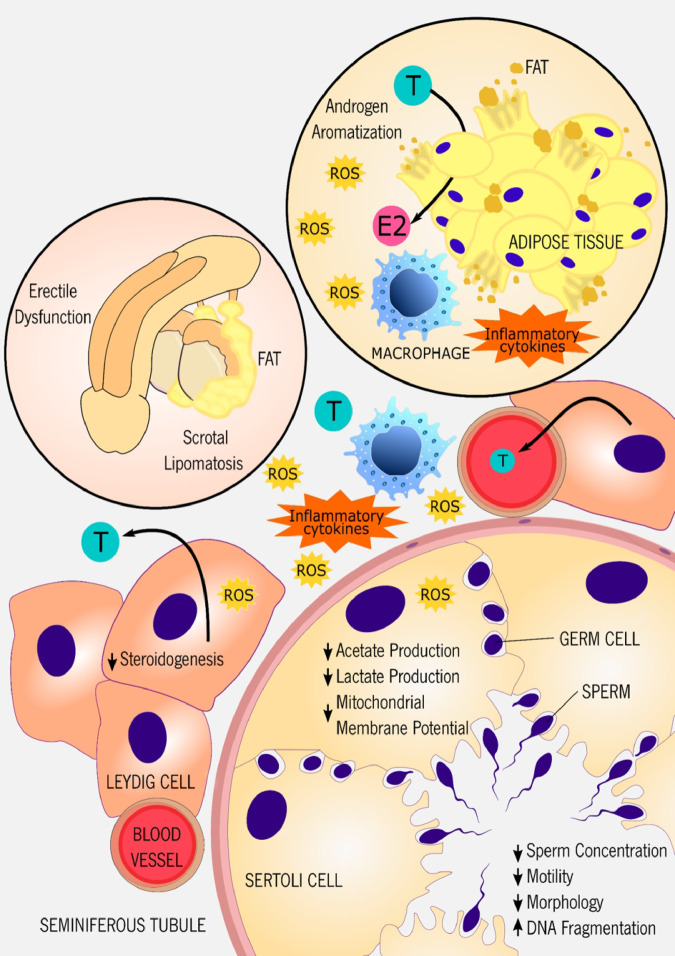
the excessive white fat deposition, a typical feature of obesity, is a threat to male reproductive function by several mechanisms. Obese men are commonly associated with an increased risk of developing erectile dysfunction and penile vascular impairment. Moreover, fat deposition on the scrotal area, a condition called scrotal lipomatosis, promotes the rise of scrotal temperature, inducing germinal atrophy and spermatogenic arrest. Testosterone aromatization into estradiol by adipocytes can induce hypogonadism (a condition characterized by low levels of androgens). The excessive lipid accumulation leads to adipocytes rupture, trigging a pro-inflammatory state. Due to its location, LCs are exposed to the ROS and inflammatory cytokines produced by the macrophages. Consequently, the steroidogenesis machinery is compromised, and the production of testosterone drops, further promoting hypogonadism. In response to the abnormal metabolic situation, SC metabolism is remodeled, decreasing both the production of lactate and acetate. Meanwhile, the mitochondrial membrane potential is also affected, threatening the ‘nursing’ of developing germ cells by SCs. E2, estradiol; ROS, reactive oxygen species; T, testosterone

There are several studies focused on obesity and classic sperm parameters (namely, concentration, motility, and morphology) with mixed results (summarized in Table 1) (29–49). While several studies showed that excess weight is associated with low semen volume and sperm motility as well as aberrant sperm morphology (29, 45, 49), other studies failed to show such a clear correlation (31, 43, 46). The observed discrepancies in the literature are likely caused by the several limitations associated with studies performed in humans. In addition, sperm function can be altered by innumerable lifestyle factors, such as smoking, alcohol consumption, and recreational drugs, which are confusing factors difficult to control in human studies. Furthermore, the use of BMI as an adiposity calculator has also been challenged (50). Obesity is also associated with the development of other complications, such as DM and oxidative stress, which can also impair sperm parameters (51, 52), promote sperm DNA fragmentation (31, 53), and induce aberrant sperm mitochondrial function (49, 53). Other comorbidities associated with obesity have also been pinpointed as responsible for the detrimental effects of obesity in sperm quality. Beyond weight gain and adiposity, there are other relevant factors associated with obesity impact in sperm quality, including hormonal dysfunction, fat accumulation in reproductive organs, accumulation of environmental toxic substances, or even inflammation and oxidative stress. Below we briefly discuss each of these factors (Fig. 2).

**Table 1:** summary of studies investigating the effects of obesity on classic sperm parameters

Author(s), year	Concentration	Motility	Normal morphology	Population
Jensen *et al.* (2004) (29)	↓	No correlation	↓	1558 young men (average: 19 years)
Pauli *et al.* (2008) (30)	No correlation	No correlation	No correlation	87 adult men
Chavarro *et al.* (2010) (31)	No correlation	No correlation	No correlation	483 men from subfertile couples
Hofny *et al.* (2010) (32)	↓	↓	↓	42 fertile obese men and 80 infertile obese men
Martini *et al.* (2010) (33)	No correlation	↓	No correlation	794 adult men
Paasch *et al.* (2010) (34)	↓	No correlation	↓	2157 men (17–67 years)
Sekhavat and Moein (2010) (35)	↓	↓	No correlation	852 men (25–50 years)
Wegner *et al.* (2010) (36)	No correlation	No correlation	No correlation	107 men attending an infertility clinic
Rybar *et al.* (2011) (37)	No correlation	No correlation	No correlation	153 men attending an infertility clinic
Shayeb *et al.* (2011) (38)	No correlation	No correlation	↓	2035 men from subfertile couples
Hammiche *et al.* (2012) (39)	↓	↓	n/a	450 men from subfertile couples
Anifandis *et al.* (2013) (40)	No correlation	↓	n/a	301 men from subfertile couples
MacDonald *et al.* (2013) (41)	No correlation	No correlation	↑	511 men attending an infertility clinic
Belloc *et al.* (2014) (42)	↓	↓	No correlation	10 665 men consulting for a semen analysis
Al-Ali *et al.* (2014) (43)	No correlation	No correlation	No correlation	2110 men attending an andrology unit
Thomsen *et al.* (2014) (44)	No correlation	No correlation	n/a	612 men from infertile couples undergoing ART
Luque *et al.* (2015) (45)	↓	↓	↓	4860 adult men (18–65 years)
Tang *et al.* (2015) (46)	No correlation	↓	No correlation	617 male infertility patients
Tsao *et al.* (2015) (47)	↓	No correlation	↓	7630 adult men
Calderón *et al.* (2018) (48)	↓	↓	No correlation	30 obese men and 10 lean men
Oliveira *et al.* (2018) (49)	↓	↓	↓	1824 men from subfertile couples

Sperm quality worsening is represented by (↓), and sperm quality improvement is represented by (↑). Not addressed parameters are represented by (n/a).

### Hormonal Dysregulation

Obesity causes whole body hormonal dysregulation. Hormones related to energy homeostasis—insulin, glucagon, leptin, glucagon-like protein-1 (GLP-1), ghrelin—have also been implicated in male reproductive dysfunction. Due to the essential role of hormones in the maintenance of glucose homeostasis, hormonal disruption affects not only signaling pathways associated with food intake control but also functions intimately related to energy homeostasis, such as reproduction (54). Leptin is a peptide hormone mainly produced by white adipocytes, which is related to adipose tissue mass and decreased food intake. In humans, leptin and sex hormones blood concentrations appear to be strongly related (55). Usually, androgens suppress leptin production (55), supporting that testosterone (T) is an important regulator of leptin. Concurrently, leptin has been found to exert important effects on gonadal organs through leptin receptors that were reported in testicular cells, including in the surface of Leydig cells (LCs) (55, 56) and Sertoli cells (SCs) (57). The excess of circulating leptin found in obese men contribute to reducing T concentrations due to impairment of LCs (58). High levels of leptin are also known to modulate SCs metabolism, decreasing lactate dehydrogenase activity (57), which has the potential to compromise male fertility. Concordantly, higher serum leptin levels found in obese infertile men were found to be correlated with abnormal sperm morphology (32).

Contrarily to leptin, circulating ghrelin levels are lower in obese than in lean men (59). Recent *in vitro* studies have reported that ghrelin can inhibit testicular secretion of T (60) while suppressing the luteinizing hormone (LH) and follicle-stimulating hormone (FSH) secretion by the pituitary (61). Ghrelin appears to carry anti-proliferative action in several cell lines, which led to the hypothesis that ghrelin can inhibit the proliferative activity of immature LCs (62). Furthermore, it avoids excess build-up of germ cells, which appears to be a crucial spermatogonia survival factor (63). Martins *et al.* (64) reported that glucose consumption and mitochondrial membrane potential of human SCs are sensitive to ghrelin levels. Those results suggested that ghrelin acts as an energy sensor for human SCs in a dose-dependent manner, modulating the nutritional support of spermatogenesis (64). Thus, it appears that ghrelin balance can contribute to the metabolic dynamics within the male reproductive axis in situations of energy deficit (54, 61).

It is also known that the incretin effect, a process that describes the increase in insulin plasma levels along with the decrease in glucagon levels, after a meal, is impaired in obese individuals (65). This evidence suggests that incretins, such as GLP-1, may be altered in obese subjects. However, the findings regarding GLP-1 levels in obesity were inconsistent and its variations have not been conclusively determined (65). Yet, some evidence suggested that leptin may exert a regulatory effect in GLP-1 secretion and that impairment of leptin regulation associated with obesity may also be associated with impairment of GLP-1 regulation (66). Although the effects of GLP-1 in male reproduction remain overlooked, its contribution to glucose homeostasis suggests that it may be important for the regulation of spermatogenesis. The expression of the GLP-1 receptor was first identified in isolated human SCs by Martins *et al.* (67). The authors also reported that exposure to GLP-1 decreased glucose consumption while increasing lactate production, in those cells. Furthermore, GLP-1 modulates glucose metabolism of SCs and is suggested to have an anti-apoptotic effect in developing germ cells (67). In addition, it decreases T secretion (68), highlighting a possible effect in the hypothalamic–pituitary–gonadal (HPG) axis. Furthermore, this hormone seems to increase gonadotropin-releasing hormone (GnRH) secretion, which suggests that it may have an important role in puberty development (69).

Hyperglycemia, which is usually found in obese men, seems to cause a decrease in sex hormone-binding globulin (SHBG) production by the liver (70). SHBG is responsible for the transport of sex hormones through the blood to the target tissue. Concurrently, with a decrease in SHBG concentration, T availability will also decrease. Hyperglycemia *per se* has long been associated with compromised male fertility. The frequency and duration of hyper- and hypoglycemic events, the degree of glucose control, age, and other factors are important to evaluate the incidence of infertility (71). However, it has been reported that poor glucose control, such as in diabetic men, is associated with impairment of sperm motility (72), sperm DNA fragmentation (73), and hormone dysregulation (74). In addition, gonadotropin secretion tends to progressively decrease in obese men (56). Overweight is also associated with increased androgen aromatization by the adipose tissue. This may result in a decrease in total and free T in serum, associated with hypogonadism (75).

### Fat Accumulation on the Scrotum

Obese individuals have an excess and abnormally distributed fat in the testis, a condition known as scrotal lipomatosis, and associated with infertility (76). The abnormal distribution of scrotal fat can form a diffuse sheet of fat with variable thickness and diffuse fat covers the cord veins and the spermatic cord. In addition, a lobular pattern of fat distribution can be found in the internal spermatic fascial tube (76). One of the main reasons why lipomatosis induces infertility is the impairment of thermoregulation in the testis of those individuals. Ideally, the scrotal sac is composed of thin skin with minimal subcutaneous fat, dense sweat glands, and scant hair distribution. These characteristics are essential for testicular aeration and heat radiation dispersion. Furthermore, vasodilation of the scrotal vessels and activation of the sweat glands are crucial for the maintenance of the testicular temperature 2–4°C lower than the body temperature (77). Excess scrotal fat increases insulation, rising scrotal temperature, and promoting testicular germinal atrophy and spermatogenic arrest. The reason behind germ cell vulnerability to heat stress lies in their higher mitotic activity. Specifically, hyperthermic testis induces germ cell apoptosis (78), autophagy (79), DNA damage, and generation of reactive oxygen species (ROS) (80). The excess of fat can compress the cord veins of the testis, being the cause of testicular ischemia, a condition characterized by the increased tension within the testis. Furthermore, the compromised blood pumping results in a venous status of the testis and testicular congestion (76) which, translates in metabolic impairment of the gonads.

### Accumulation of Environmental Toxic Substances

Some environmental toxins, usually agriculture pesticides or industrial compounds, act as endocrine disruptors (81). Indeed, obese individuals are at higher risk to suffer from the action of these disruptors since a large portion of them are liposoluble (82) and tend to accumulate in fat. However, the interaction among adipose tissue, environmental toxins, and male fertility remains to be elucidated. Still, the presence of organochlorines, a class of toxins usually found in pesticides (83), has already been reported in the seminal fluid of men. Some studies have also evaluated the impact of these compounds on sperm quality parameters. The exposure to some organochlorines is associated with decreased sperm counts (84), while aromatic hydrocarbons, another class of toxins, are reportedly described as major contributors for the dysfunction of sperm parameters (85). Although the presence of liposoluble toxins in the testis is likely one of the ways by which obesity induces infertility in males, this topic has been overlooked over the years (86).

### Inflammation and Oxidative Stress

The balance between death/regeneration of developing germ cells is essential for the maintenance of spermatogenesis. ROS are important modulators of several apoptotic signaling pathways, including the p38 MAPK (*Mitogen-Activated Protein Kinases*) pathway. Damaged germ cells produce ROS, which can then activate the p38 MAPK pathway thus starting the apoptotic process (87). This process highlights the importance of ROS in the regulation of testicular germ cell populations, under stress conditions. ROS produced by testicular macrophages also mediates steroidogenesis. The close physical association between this cell and LCs suggests that they are functionally related. Indeed, ROS inhibit cholesterol mobilization to mitochondria in LCs, which is a crucial step for steroidogenesis. Concurrently, the degradation of the steroidogenic machinery is promoted by ROS and other inflammatory cytokines (88). Nevertheless, mature spermatozoa are highly susceptible to oxidative stress due to their limited amount of antioxidant machinery and cytoplasm (89). Obese individuals have a high metabolic rate, to restore the energetic homeostasis of the body. As a result, oxygen consumption rates increase, leading to ROS overproduction through the mitochondrial respiratory chain (90). This situation is aggravated by the permanent state of inflammation caused by the rupture of adipocytes due to triglyceride saturation (15). Consequently, macrophages invade the tissues, where pro-inflammatory cytokines are released (91). In the testicular environment, ROS is a major contributor to sperm cell dysfunction, inducing DNA damage and compromising cell membrane integrity in spermatozoa (89). Also, it has been observed that a higher amount of ROS in obese men causes altered DNA methylation, meaning that methylation changes in sperm may alter the embryo development and phenotype of the offspring (92). This close relationship between inflammation and oxidative stress in the testis deserves special merit, particularly to understand the subfertility and infertility associated with overweight and obesity.

## Direct Effects of Western Diet in Spermatic Quality

As human populations became more urban, fiber-rich diets were replaced by sugar and fat-rich diets. This change in food habits is often explained as those diets are affordable, readily available, and palatable. Along with that, our social environment invites to the consumption of large-sized meals while also promoting a sedentary lifestyle (93). Nevertheless, the exact role of fat-rich diets on the development of obesity is still controversial. It is tempting to summarize it as fat-rich diets, along with a lack of exercise, ultimately leading to a positive energy balance, since energy intake is higher than energy expenditure, and thus weight gain. However, the metabolic significance of that weight gain depends on several factors, including the special distribution of fat, the type of fat, and the dysregulation associated with it. Depending on all those factors, distinct health effects can be induced. For instance, unhealthy diets rich in trans and saturated fats, red processed meat, refined grains, and sweets are associated with poor semen quality (94). Meanwhile, healthy diets, rich in vegetables, fruits, whole grains, and seafood have been associated with better sperm parameters and a lower likelihood of having abnormal sperm concentrations, total sperm count, and motility (95). However, this may be more complex than initially thought as some papers report that overweight and obesity are not associated with loss of sperm quality (37, 43) Curiously, the hormonal disruption of the HPG axis was reported by the large majority of these studies (30, 37, 43). This hypothesis proposes that the main effect of obesity on male fertility is on libido and sexual performance rather than sperm quality parameters in itself. Furthermore, it is proposed that the detrimental effect of obesity on spermatic quality can only be observed in morbid-obese individuals (30). Notwithstanding, spermatogenesis requires a large variety of building blocks during the formation of spermatozoa. This means that, along with the metabolic impairments associated with overweight/obesity, the food that individuals consume will have a direct impact on sperm production and will be reflected in the spermatic quality.

### The Impact of Sugar-Rich Diets on Sperm Parameters

Some centuries ago, sugar was a delicacy only available for the wealthiest. The globalization of sugar started when the Portuguese began the exploitation of sugarcane and sugar refinery, in Brazil, through the implantation of a slave-based economy, during the late 15th century. The consumption of sugar raised to mass consumption around the mid-17th century due to the industrial revolution and, nowadays, sugar is one of the cheapest foods in the world, costing, on average, <10 cents per pound (≈450 g), or 1800 calories of sugar (93). Furthermore, sugar is a powerful flavor booster, meaning that sugar-rich foods tend to be more palatable. The relationship among sugar consumption, body weight gain, and the incidence of obesity is widely acknowledged (1, 96). Moreover, the consumption of high doses of sugar is associated with the development of complications normally associated with obesity, such as metabolic dysregulation, cardiovascular diseases, and T2D (96, 97). Until now, it remains unclear whether the damage to sperm is attributed to local effects from hyperglycemia or to disruption of the HPG axis. Uncontrolled hyperglycemia was associated with lower leptin secretion, promoting a decrease in LH secretion (98). Both hormones play crucial roles in the integration of fat cells signals in the reproductive system. Indeed, the drop in leptin levels has been associated with reducing numbers of sperm within the seminiferous tubules and shrunken LCs in male mice (99). Another study performed in mice revealed that long-term hyperglycemia leads to significant testicular dysfunction and decreased fertility potential (100). After 6 months of induced hyperglycemia, it was observed fewer LCs. At the same time, it was also detected fewer LH receptors, leading to an accentuated fall of T levels in diabetic animals. As a consequence, the seminiferous tubules from these animals presented depletion of germ cells and, structural abnormalities between Sertoli–Sertoli junctions were found (100). In humans, hyperglycemia has been associated with an increase in sperm DNA damage (73). This factor may be related to poorer embryo quality and implantation success in couples where the male suffers from DM (101). Alternatively, some contaminants, such as bisphenol A and phthalates, were identified in processed sweets and sugar-sweetened beverages. The presence of phthalates metabolites in the urine was significantly associated with decreased sperm motility, sperm DNA damage, and sperm aneuploidy (102). These studies highlight that beyond the direct effect of sugar-rich diets, we must take into consideration the hormonal dysregulation induced and the presence of contaminants that can mediate deleterious effects on the reproductive system and/or spermatic quality.

### The Impact of Fat-Rich Diets on Sperm Parameters

Almost all dietary fats are triacylglycerols (composed by three fatty acids molecules bound by a glycerol molecule) can be categorized into four distinctive classes: saturated fatty acids (SFA), monounsaturated fatty acids (MUFA), polyunsaturated fatty acids (PUFA), and trans fatty acids (TFA). This classification is based on the presence, absence, number, and type of double bonds which alter the biochemical proprieties of the fatty acid molecule (103).

The first animal products consumed by men were derived from wild animals, whose fat content and quality in meat vary with species, age, sex, and season. In wild animals, MUFA and PUFA constitute the large majority of triacylglycerol present in the edible meat, since the SFA reservoirs are depleted during most of the year. Because of the seasonal depletion of SFA, pre-Neolithic diets were rich in MUFA and PUFA, which are known to prevent the onset of T2D and cardiovascular diseases (104). Some centuries later, cattle started to be fed with grains and seasonal depletion of SFA stopped. This significantly decreased the animal life expectancy (only 24 months until slaughter), while excessively increased SFA content in the edible meat (104). Ruminant animals are also natural producers of TFA, due to intestinal bacterial enzymes which catalyze the partial hydrogenation and/or isomerization of *cis*-unsaturated fatty acids. Thus, dairy products are also rich in TFA (105). Along with this, western-diet is known for the high consumption of processed products, rich in TFA due to the hydrogenation vegetable oils (106).

Spermatozoa fatty acids are predominately composed of SFA (around 60%), however, their plasma membranes are predominantly composed of phospholipids and a significant amount of PUFA. TFA account, on average, for <1% of the total fatty acids content of sperm (107). Chavarro *et al.* (107) reported that total sperm TFA content was inversely correlated with sperm concentration while total sperm PUFA content was positively correlated with that parameter. The intake of TFA was also inversely associated with sperm concentration. A proposed hypothesis is that TFA can interfere with the incorporation of PUFA into sperm membranes, consequently affecting spermatogenesis (108). Furthermore, the intake of TFA was reported to decrease total T concentration and lower testicular volume, affecting, therefore, the HPG axis (109). In boars, the SFA content of sperm was negatively correlated with sperm quality. In opposition, PUFA sperm content was positively correlated with motility, viability, and normal morphology (110). In humans, the intake of SFA has also been inversely correlated with sperm concentration and total sperm count (111). Furthermore, the high intake of SFA and TFA represents a risk factor for asthenozoospermia (the medical term for reduced sperm motility) (112). Alternatively, it is relevant to note that anabolic sex steroids are administrated to cattle for growth promotion. Although the European Union banned this practice in 1989, it is still practiced in other parts of the world (113). The hormone levels in edible tissues are higher in treated animals than non-treated animals (114, 115). Afeiche *et al.* (114) reported that intake of processed red meat was associated with a decreased sperm count in young males. Another study revealed that the consumption of processed meat during pregnancy is associated with lower sperm concentration among the male offspring (116). Furthermore, men who consume highly processed red meat were associated with a higher risk of asthenozoospermia (117). However, due to the paucity of data about this topic, further studies are needed to establish a solid correlation between meat ingestion and sperm parameters, particularly concerning meat origin and how it can induce metabolic signatures in sperm.

## Metabolic Signatures Induced by Diabetes and Prediabetes in Testis

T2D is often associated with overweight and obesity, although it is not limited by it. In fact, it is reported as early as the 1980s that there are individuals with normal weight, considering BMI, who are ‘metabolically obese’ (118). Despite that, overweight and obesity, associated with increasing adiposity, are risk factors for the development of a phenotype coined as prediabetes and that can rapidly progress to T2D (97, 119). Prediabetes and T2D are characterized by persistent hyperglycemia and are caused by either an increasing degree of cellular insulin resistance or insufficient secretion of insulin in response to glycemia, by the pancreatic β-islets. The dysregulation of glucose homeostasis, also known as glucose metabolism or whole-body metabolism, is the main driving force toward metabolic reprograming in different tissues. The persistent elevated glycemia levels are also related to T2D comorbidities, particularly microvascular (retinopathy, nephropathy, neuropathy) and macrovascular (coronary artery disease, myocardial infarction, stroke, congestive heart failure, and peripheral vascular disease) disorders (120). Those disorders are further responsible for other typical complications found in diabetic patients, such as glaucoma, chronic kidney disease, poor wound healing, and increased infection risk (which often leads to amputation) (121). The reproductive system is also affected by T2D-related comorbidities. Besides microvascular disorder associated with erectile dysfunction, diabetic men often suffer from retrograde ejaculation and subfertility. This is not surprising, as reproductive function requires proper nutritional support, and thus is sensitive to nutritional status and whole-body energy balance (5, 8, 122). Recently, more evidence has been published regarding the metabolic changes induced by a hyperglycemic state in male reproductive function (122–126). Besides, there is a growing concern about the metabolic signatures of hyperglycemia in the male gamete, as a potential epigenetic factor capable of modulating the predisposition for the onset of metabolic disease on the offspring (127, 128).

### Glucose Homeodynamics and Spermatogenesis

In mammals, spermatogenesis is initiated during puberty. By the onset of this developmental phase, specialized neurons in the hypothalamus increase the frequency of the pulsatile secretion of GnRH, which is transported via the hypothalamic-hypophyseal portal system to the anterior hypophysis (also known as adenohypophysis), where it is recognized by GnRH receptors expressed by gonadotropes. In response, these cells synthesize and secrete into the bloodstream the gonadotrophic hormones, FSH and LH. In males, LH stimulates the production and secretion of androgens (e.g. testosterone and dihydrotestosterone) by LCs, which in turn are responsible for secondary sexual characteristics (129). The main target of FSH is the SC (130), which acts as a central mediator in the differentiation process of germ cells, known as spermatogenesis (131).

The onset of the GnRH pulse is one of the earliest known effects of metabolic variables. In humans, the onset of puberty varies in a board range in the function of climate, race, and gender (132). However, regardless of any other factor, undernutrition delays the onset of puberty, while overweight and childhood obesity anticipate it (133). Although this effect is not directly related to gamete epigenetic signature, in males, an early onset of puberty caused by overnutrition will mean that the first spermatogenic cycles will be completed in a high-energy environment, capable of inducing irreversible changes in testicular metabolism (134). Even though spermatogenesis is the differentiation process of spermatogonia into mature spermatozoa, SCs are the main players in this process, providing nutritional, material, and structural support to differentiating germ cells. In fact, due to the blood-testis barrier, differentiating germ cells are secluded within the adluminal space of seminiferous tubules and confined to microenvironments created by SC cytoplasmatic protrusions. Therefore, the metabolic needs of germ cells depend on SC function, which in turn is sensitive to the metabolic status of the subject or animal. For instance, in SCs, the *Ins2* (insulin-2) gene expression is regulated by *Rhox* family members, with a direct impact of insulin signaling (135). Rhox is a homeobox gene sequence, which codes for a transcription factor that modulates the expression of target genes encoding proteins involved in processes relevant to spermatogenesis. Insulin has also an important regulatory role over the reproductive axis. Akita rats, a model for type 1 diabetes (T1D), were demonstrated to resume spermatogenesis after injection of external insulin doses, by the restoration of the normal blood concentrations of gonadotropins (136). High-energy diets were shown to induce a pre-diabetic state in rodents causing shifts in the glycolytic metabolic profile of SCs (126). The authors further elicited that this effect is linked to T deficiency, caused by progressive stages of DM, ultimately translated into metabolic reprograming of SCs (137). Taken together, those studies report the effects of SC metabolic reprograming over sperm parameters in rodents (126, 137). In sum, glucose homeodynamics during spermatogenesis causes metabolic signatures that can be further imprinted in sperm and open the possibility of changing the (epi)genetic information that is passed to the next generations.

### Glucose Dysfunction, Sperm, and Reproductive Parameters

The impairment of sexual function is not regarded as one of the most immediate comorbidities of DM. Nevertheless, as the global prevalence of DM is expected to increase in the upcoming years, and, in the case of T2D, at progressively lower ages, there is a growing concern about the prevalence of the disease in men on reproductive age (97, 119). Moreover, as the reproductive function is dependent upon the individual’s nutritional state, the symptomatic loss of glycemic control observed in DM is expected to produce negative outcomes in sperm and reproductive parameters (8, 122, 126). Indeed, ejaculated sperm from men suffering from T1D and T2D presents poorer quality parameters (lower motility, viability, and DNA integrity) than normoponderal men (138). In their study, Roessner *et al.* (138) relate the deterioration of sperm parameters with an overactivation of apoptotic signaling in sperm cells, such as the Casp3 (caspase-3) programed cell death pathway. In couples followed due to fertility problems, the success rate of embryo transfers resulting from assisted reproductive techniques (ART) is lower when the male is diagnosed with DM (101). This effect is also reported in couples who did not seek medical advice toward ART: in couples where the male partner was affected by DM, the time-to-pregnancy was longer, independently of sexual frequency (139). The molecular mechanisms remain somewhat unknown, but several animal models support those observations and allow the study of some mechanisms. In one of the earliest studies on this phenomenon, Seethalakshmi *et al.* (140) observed lower sperm counts and motility in streptozotocin diabetes-induced rats, concomitantly with elevated seminal fructose, lower gonadal weight and lower levels T, LH, and FSH. After insulin treatment, all those parameters were recovered to control-like values (140) illustrating that glucose dysfunction was responsible for the detected reproductive problems. Rats fed with a fat-rich diet develop a diabetic or pre-diabetic etiology (increase adiposity and hyperglycemia) which induces lower sperm quality but improvements in metabolic function and sperm parameters can be achieved after diet, exercise or a combined diet/exercise plan (141). Exercise has been prescribed as a tool to improve glycemic control in humans, by improving overall glucose utilization at the cellular level with positive results (142, 143). However, volume, intensity, and frequency of physical exercise must be dosed accordingly to the specificities of the diabetic patient (144). Therefore, other metabolic solutions have been explored, such as nutritional control and functional foods. For instance, vitexin, and apigenin flavone glucoside present in some herbal infusions was shown to improve reproductive behavior, sperm and fertility parameters in streptozotocin-induced diabetic mice (145). Similarly, the substitution of water by white tea has been demonstrated to improve sperm parameters in pre-diabetic mice (146). This effect was further explored *in vitro*, with human SCs, supplemented with epigallocatechin-3-gallate (EGCG), the most abundant tea catechin (147). At a concentration of 50 μM, EGCG increased glucose and pyruvate, maintained lactate production whilst decreasing oxidative damage and mitochondrial membrane potential of human SCs (147). These examples illustrate how nutritional variables can reprogram SCs metabolism, which will ultimately affect spermatogenesis.

### Small-RNAs and Epigenetic Mechanisms May Also Be Affected by Metabolic Dysregulation

**Figure 3:**
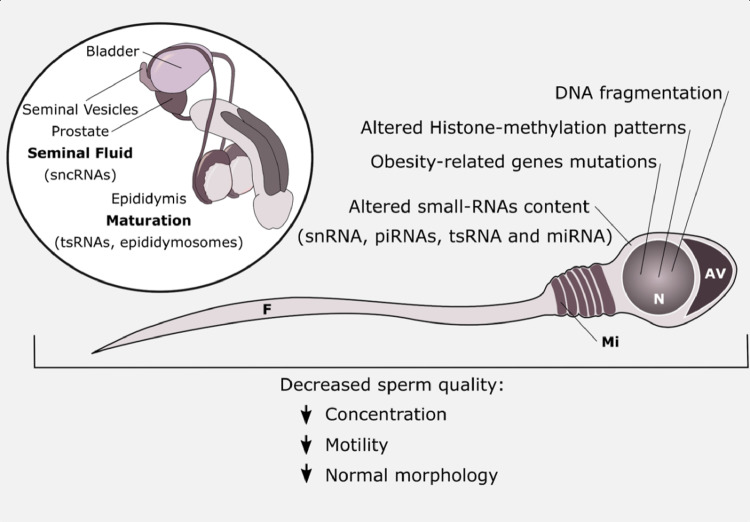
epigenetic signatures carried by sperm that are affected by metabolic profile. Even after completing spermatogenesis, sperm epigenetic signatures are modulated through its transit in the male reproductive tract. For instance, the maturation process at the epididymides and the seminal fluid resulting from seminal and prostate secretions modulate the overall epigenetic cargo of spermatozoa. Obesity and T2D interfere with the relative abundance of different epigenetic factors, which are later expressed in phenotypic alterations such as lower sperm concentration, lower motility, and increased prevalence of abnormal spermatozoa. AV, acrosomal vesicle; F, flagellum; Mi, mitochondria; N, nucleus

In recent years there has been increasing awareness regarding the epigenetic contents carried by spermatozoa. Notably, there is a growing number of published papers on sperm epigenetic regulation by the action of small-RNAs (sncRNAs, piRNAs, tsRNA, miRNAs), DNA methylation, and chromatin dynamics (post-translational histone modification and protamination) (127, 148, 149). piRNAs (Piwi-interacting RNA) is the largest class of snRNAs. They form RNA–protein complexes through interactions with Piwi-subfamily Argonaute (RNA-induced silencing complex) proteins. They are involved in epigenetic silencing, post-transcriptional silencing, and regulation of gene expression in germ cells. tsRNA (tRNA-derived small RNAs) interfere with the tRNA functions. There is also extensive literature supporting the essential role of RNA species for germline progression [for a review, Lehtiniemi and Kotaja (150)]. Presently, there are a plethora of small-RNAs reportedly found in mammalian testes. Some of them, as the miR-34 and let-7 miRNA families (151) are sensitive to metabolic variables, namely T2D (152). Another study, in mice, showed that piRNAs were crucial spermatogenic regulators in meiotic cells. Herein, the authors reported the meiotic piRNA population can silencing specific mRNAs, regulating by this way the progression of meiosis (153). DNA methylation in spermatozoa is also affected by the metabolic profile. The DNA methylation pattern (which included *FTO*, *MC4R*, and other metabolic genes) found in sperm from men who underwent bariatric Roux-en-Y gastric bypass surgery was different from the pattern found in sperm from pre-intervention cohorts (154). In addition, the differences were observed as early as 1 week after surgery, but even more evident after 1 year (154). The Roux-en-Y is often called bariatric-metabolic surgery because it not only acts to reduce weight but it is also effective in dampening insulin resistance (155). Despite this, it is not possible to unquestionably postulate whether the effects observed by Donkin *et al.* (154) are a result of improved glycemic or weight/adiposity reduction. Sperm chromatin dynamics are also related to methylation, as both mechanisms regulate the accessibility to DNA sequences (genes) for further transcription. Besides, the enzymes responsible for DNA (de)methylation and histone modification are sensitive to metabolic variables, and local availability of energy substrates and cofactors (156). Herein, it is possible to include the sirtuin family of deacetylases, which nuclear members are known to control the epigenetic state of chromatin in an NAD^+^-regulated manner (156). Although chromatin is majorly condensed around protamines, the portions of histone-bound DNA in germline cells seem to bear essential information, notably imprinting genes, for adequate spermatogenesis (157), fertilization (158) and early embryo development (159). These changes, induced by metabolic dysfunction, can be further passed to the next generations (Fig. 3).

## Paternal Transgenerational Effects of Metabolic Dysfunction

The epigenetic signatures carried by the spermatozoon that manages to fuse with the oocyte are inherited by the offspring (160). The extent of epigenetic signatures of spermatozoa that influence the outcome of the offspring metabolic status can start from the early developmental phase to birth and even later into adulthood (128). The findings in this field have shaken the very basis of Evolutionism, prompting a new glance of the role of Physiology over it, as emphasized by Noble *et al.* (161) in their Editorial for ‘*The Journal of Physiology*’. Epigenetic inheritance was a controversial theory because it was considered that epigenetic signatures were reset after gamete fusion to permit zygote’s totipotency (162).

For a long time, it was assumed that sperm could not carry potential epigenetic factors into the oocyte, especially because of protamination, demethylation, and the small cytoplasmatic volume. However, over the last decades, this perspective was drastically changed. Protamination replaces canonical histones with specific sperm protamine proteins after meiosis, an essential set for DNA compaction in mammal (163). Protamines can compact paternal DNA to a millionth of its original volume, or six times denser than in somatic cells (164), which is crucial for sperm head size and dynamics (165). It was thought that this process would remove all epigenetic modification from the sperm, hence epigenetic inheritance was primarily attributed to the mother (166). Nowadays, it is known that ∼5 to 15% of sperm chromatin remains nucleosome-bound (158, 166). Hammoud *et al.* (167) reported that the retained nucleosomes are significantly enriched in imprinted gene clusters, microRNA and other signaling factors. Therefore, it was postulated that retained histones could contain modifications that play an epigenetic role in embryonic regulation (167). Further studies consolidated this concept. A gene ontology analysis revealed that genes carried by histones were associated with metabolic and development processes (168). Furthermore, even though the position of the histone is unaltered in both spermatozoa from lean or obese men (168), DNA methylation patterns are markedly different (154).

Methylation, notably on CpG islands (repetitive DNA sequences containing C-G nucleotides regulating gene expression) is erased in the primordial germ cells during mammal embryonic development (169). There is evidence now that this demethylation is not total: it is reduced by 70% on the first phase (170) and up to a minimum of 8% in humans after a second demethylation cycle (171). In fact, paternal obesity was correlated with altered methylation status of multiple imprint regulatory regions from the offspring genome. The authors measured the methylation percentage in the extracted DNA from the umbilical cord blood leucocytes of 92 newborns, born from parents defined as obese through the BMI scale. These results highlight how imprinted instabilities may be transmitted to the next generation, although the molecular mechanisms by which it occurs were not fully elucidated (172). Genes involved in neurological diseases and metabolic regulation are epigenetic hotspots in gametes (154). Interestingly, among the genes reported to be differently methylated in spermatozoa from obese and lean individuals, there is a group of genes that are potential carriers of polymorphisms associated with onset-obesity. Some genes from this group are the fat mass and obesity (*FTO*), melanocortin-4 receptor (*MC4R*), and the transmembrane protein 18 (*TMEM18*) (154).

Paternal epigenetic inheritance was also considered unlikely as the spermatozoon was believed to simply carry genomic information, and it would not even contribute with mitochondria to the zygote. Moreover, even if it carried any other inheritance factors on its cytoplasm, the scale difference between it and the oocyte’s cytoplasm would render them insignificant for the zygote’s outcome. For some decades now, it is known that sperm is completely incorporated in most mammalian oocytes (173), and the first cases of mitochondrial heteroplasmy in humans were recently reported (174). Moreover, some epigenetic inheritance mechanisms have been exclusively identified in mammalian male gametes. For instance, contrarily to protamine regions of the sperm genome, sperm histones are not replaced by maternal nucleosomes after gamete fusion and contribute to zygote’s chromatin (158).

Although the mechanisms involved are not fully understood yet, the offspring of obese individuals are at greater risk toward the development of metabolic disorders, later in life. For instance, the mortality rate is increased in adult offspring of obese mothers, especially from cardiovascular diseases (175). Some studies have also demonstrated the potentially harmful effects of paternal food habits in the offspring health. The *Överkalix* study (176) and the ‘The Dutch Famine Birth Cohort Study’ (177), both highlight the influence of parental food habits in the development of metabolic diseases in the offspring. Yet, animal models have provided the most evidence on the mechanisms underlying paternal transgenerational effects. The increase in metabolic diseases is expected to be accompanied by an increase in infertility. More people will be seeking fertility treatments and the levels of anxiety and depression associated with involuntary childlessness are expected to rise in the coming years (178). Therefore, it is essential to understand the molecular pathways by which metabolic disorders are inherited by the offspring of overweight/obese parents, and how the development of metabolic diseases could be prevented in predisposed individuals.

### Evidence from Animal Studies

Animal studies are the best suited experimental model to study the transgenerational effects of the signatures induced by metabolic profile in male gametes. It is simply not possible timewise, nor is it ethical, to study those effects in a human population. Table 2 synthesizes several examples of studies where mammalian models were used (179–188). One of the earliest works on the function of mammalian sperm microRNAs revealed the role of miR-34c on the first zygote division, in mice (189). Rats fed by a fat-rich diet overexpress let-7c miRNA on sperm cells, a phenotype also observed in the adipose tissue of their offspring (186). Moreover, the female offspring showed a metabolic reprograming, related to shifts in let-7c expression and subsequent transcriptomic shift of its targets, causing glucose intolerance and resistance to diet-associated weight gain (186). Another breakthrough study, by Grandjean *et al.* (183), showed how a miRNA can induce a metabolic disease phenotype *per se*. In this work, miRNA miR19b was injected in naïve mice zygotes, inducing a metabolic phenotype in resulting mice similar to the changes verified after diet-induced metabolic disease (183). These results need further confirmation in humans but are the basis of a novel concept regarding the inheritance of metabolic diseases.

**Table 2:** mammalian studies on paternal transgenerational inheritance of phenotypes associated with altered metabolic profiles (diet, metabolic disease, exercise)

Author(s), year	Metabolic trigger	Species, generations	Effects on offspring	Proposed mechanism
Carone *et al.* (2010) (179)	Paternal low-protein diet (F0)	*Mus musculus*, F0 + F1	Increased expression of genes related to lipid and cholesterol biosynthesis in liver, reduced cholesterol esters	Hypermethylation of *Ppara* gene
Ng *et al.* (2010) (180)	Paternal fat-rich diet (F0)	*Rattus norvegicus*, F0 + F1 (female)	Pancreatic β-cell dysfunction	Inhibition of gene expression in pancreatic islet cells, hypomethylation of *Il13ra2* gene
Guth *et al.* (2013) (181)	Parental physical activity (F0)	*M.musculus*, F0 + F1 + F2	Changes in expression of metabolism-related genes. F1 females: lower body height, decreased omental fat. F2 females: increased glycemia	Germline inheritance of gene expression patterns
Wei *et al.* (2014) (182)	Paternal prediabetes (F0)	*M.musculus*, F0 + F1	Glucose intolerance and insulin resistance	Changes to sperm methylation pattern
Grandjean *et al.* (2016) (183)	Western-like diet (F0), RNA injection (F1)	*M.musculus*, F0 + F1, F1 (one-cell embryos)	Glucose intolerance and insulin resistance	Cytoplasmatic injection of sperm-borne miR19b into zygotes
Chen *et al.* (2016) (184)	Paternal fat-rich diet (F0)	*M.musculus*, F0 + F1	Impaired glucose tolerance and insulin resistance. Changes in gene expression of metabolic pathways in embryos and pancreatic islets	tsRNA delivery by epididymosomes to spermatozoa
Cropley *et al.* (2016) (185)	Paternal obesity and prediabetes (F0)	*M.musculus*, F1 + F2 + F3	Impaired glucose and lipid metabolism. (F1: after dietary challenge; F2: without dietary challenge)	Changes to sperm content in sncRNA, notably tsRNA
de Castro Barbosa *et al.* (2016) (186)	Paternal fat-rich diet (F0)	*R.norvegicus*, F0 + F1 + F2	F1: reduced body weight and pancreatic β-cell mass. F1 and F2 females: glucose intolerance and resistant to weight gain by fat-rich diet	Alterations in methylation pattern of spermatozoa and in the expression of let-7c miRNA
Murashov *et al.* (2016) (187)	Paternal physical activity (F0)	*M.musculus*, F0 + F1	Greater susceptibility for negative effects of fat-rich diets	Changes in methylation profile and microRNA content of spermatozoa
Sharma *et al.* (2016) (188)	Paternal low-protein diet (F0)	*M.musculus* and *Bos taurus*, F0 + F1	Overexpression of squalene epoxidase gene (cholesterol biosynthesis) in liver.	tRNA fragments delivery by epididymosomes to spermatozoa, decreased miRNA let-7c expression

### Human Studies

Transgenerational studies in human populations present significant challenges due to contingencies of time and variable control. Nevertheless, there are two special cases where a human population and its offspring were described thoroughly, providing up to this day new data for retrospective studies on the effects of metabolic conditioning on the health outcomes of the offspring. Data from the late XIX and early XX centuries, from the famous *Överkalix* study, revealed the consequences of the opposite stimuli, i.e. excess food supply, in a Swedish male population (176). This study was based on a cohort of 320 individuals born in 1890, 1905, and 1920 in the parish of *Överkalix* in northern Sweden. Thanks to the record of local communities, it is known the food availability of which year during the 19th and 20th centuries. This allowed the authors to estimate food availability during children slow growth period (SGP) (the period before the prepubertal peak in growth, 8–10 for girls and 9–12 for boys). Interestingly, sons of fathers who experience poor food availability during SGP were protected against cardiovascular death. This tendency was also found for grandsons of paternal grandmothers who were exposed to crop failure during their SGP. Grandsons of paternal grandfathers who were exposed to crop failure during their SGP appeared also protected from diabetes as a cause of death. As the authors highlight, the development of cardiovascular diseases and diabetes are also related to children's and grandchildren own nutrition, social environment, and adult life circumstances, which none were taken into consideration in this study. However, these results highlight the sensitivity of the SGP for the capture of environmental information, and gene expression alteration, which can then be transmitted to the next generation through the male line (176). Another study, based in the same period and parish of *Överkalix*, revealed that food availability during grandfathers and grandmothers’ SGP was related to grandsons and granddaughters mortality, in a sex-specific matter (190). Grandsons relative mortality increased (*P* = 0.009) when paternal grandfathers had experienced a good food supply during SGP. Similarly, granddaughters relative mortality was two-fold higher when paternal grandmothers experience a good food supply during SGP. The opposite effect was found for grandparents who experienced a poor food supply during SGP (190). The same study also evaluated the effects of father’s mid-childhood smoking on the offspring BMI. For this, a questionnaire was done to soon-to-be fathers (April 1991 to December 1992) about their tobacco habits. The authors found that fathers with earlier onset smoking habits were more likely to smoke during conception, which was associated with increased BMI in sons at the age of 9 (191). These results highlight a sex-specific transgenerational effect of paternal grandparents to the relative mortality of grandsons and granddaughters, specifically. Similarly, the transgenerational effects of father mid-childhood smoking habits on his son BMI at 9 years old reflect the importance of SGP for the capture of environmental information (190). Most importantly, these studies highlight that a male-line transgenerational response system exists in humans.

The second grand case report was provided by ‘The Dutch Famine Birth Cohort Study’ (177), as a result of observations collected from the Dutch population after World War II Dutch Famine. Indeed, this is a rare case where an advanced (high developmental index) society, where medical care is readily available and proper records are made, was affected by great stress. Thanks to medical records from this time and their descendants, it is now possible to relate epigenetic traits modulated by dietary behavior (nutritional restriction) and inheritable characteristics. Pregnant, malnourished women during this period, gave birth to underweight children who would develop metabolic complications later in life (192–194). Notably, the authors of those studies report a higher incidence of obesity and T2D (and cardiovascular disease) in this population comparing to cohort populations. These studies, analyzed nowadays with current knowledge, clearly support that there are metabolic signatures induced by diet.

None of these human studies can provide any data regarding the molecular mechanism involved in the transgenerational effects of paternal habits and offspring health, highlighting the importance of animal studies to explore these molecular mechanisms.

## Conclusions

The health costs of our societal success are becoming progressively clear. In developed countries, independently of its geographical context, disease outbreaks caused by contagious diseases are rare nowadays and are accounted for by a low death-toll every year. However, this success demands a fast pace which often pushes individuals in adopting unhealthy lifestyles, such as poor food habits and sedentarism. Therefore, a new pandemic rose: the fat pandemic. Obesity, overweight and associated comorbidities (most notably DM) have promoted noncommunicable diseases to the main cause of death and, years of health loss, in developed countries (96). Despite several efforts to tackle the increasing prevalence of obesity, overweight, and DM worldwide, the WHO predicts its prevalence to keep this trend for the upcoming decades (97, 119). The reproductive function relies on a tight nutritional and hormonal balance; therefore, it is sensitive to diet variables and global nutritional status. Fat-rich diets can affect the reproductive outcomes of men as shown in recently published clinical and biochemical evidence. Several studies even have gone further to point toward a metabolic reprograming via transgenerational effects in the male lineage, which might trigger a predisposition for the onset of obesity and related comorbidities on the offspring.

The present data shows the urge for further investment in the biochemical mechanisms correlating fat-rich diets and male fertility outcomes. From all reported cases of infertility, one-third of them is still considered idiopathic, but it is reported that the success rate is lower if the male is overweight (195). The nutritional interventions have retrieved contradictory results so far, thus it is important to address which individual characteristics promote a positive reproductive response to those methodologies. The interaction of metabolic variables, as homeostasis but also at the reproductive tissue, need further description and are likely influenced and influencers of the endocrine balance. Yet, one of the key aspects in future research on this field is the transgenerational effects of nutritional variables, via the male gamete, and the inheritance of non-genetic factors.

Recent research on the inheritance mechanisms reveals a new level of complexity. Rather than considering each gamete fusion as a full biological ‘reset’ that would virtually guarantee the ideal survival chances for the offspring (and disregarding genetic inheritance), there is now evidence that environmental and behavioral variables may also be determinant in the health outcome of the next generations. These recently unknown mechanisms have been critical in evolutionary processes, but in our present, resourceful environment, they may pose a threat to our future as a species. The more is known about the impact of metabolic profile over the signatures carried by (male) gametes, and their effects on the health of descendants, the more awareness about healthy food habits grows. Generally, individuals give more primacy to their offspring health than to their own health. Thus, research on this subject rises as a new hope to curb the ‘fat epidemic’.

## Funding

This work was supported by the Portuguese Foundation for Science and Technology (SFRH/BD/128584/2017 to L.C., IFCT2015 and PTDC/MEC-AND/28691/2017 to M.G.A., IFCT2015 to P.F.O.), and UMIB (UID/Multi/00215/2019) co-funded by FEDER funds (POCI/COMPETE 2020) and by the Portuguese Society of Diabetology (GIFT research grant to M.G.A.).

*Conflict of interest statement*. The authors declare that there are no conflict of interests.
